# Etiology, Outcomes, and Complications of Total Hip Arthroplasty in Younger Patients: A Nationwide Big Data Analysis

**DOI:** 10.3390/jcm13154535

**Published:** 2024-08-02

**Authors:** David Maman, Linor Fournier, Yaniv Steinfeld, Yaron Berkovich

**Affiliations:** 1 Carmel Medical Center, Technion—Israel Institute of Technology, Haifa 2611001, Israel; linorfournier@gmail.com (L.F.); yaron.berkovich@gmail.com (Y.B.); 2 Mount Sinai Hospital, Toronto, ON M5G 1X5, Canada; yanivsteinfeld@gmail.com

**Keywords:** total hip arthroplasty, under 55 years old, postoperative complications, clinical outcomes, NIS

## Abstract

**Background:** This study investigates the rising trend of total hip arthroplasty (THA) in patients under 55 years old, commonly referred to as “younger” THA patients. Traditionally a procedure for older adults with osteoarthritis, THA is increasingly performed on younger patients. **Methods:** Using data from the Nationwide Inpatient Sample (NIS) from 2016 to 2019, we analyze the factors driving this trend, including the causes of hip issues, patient characteristics, and coexisting medical conditions. The study examines in-hospital mortality, length of stay, post-surgical complications, and hospitalization costs for 231,630 THA patients aged 18–54.9, identified using ICD-10 codes. **Results:** Statistical analysis revealed that younger patients (aged 18–34.9) had higher rates of chronic anemia, inflammatory bowel disease, sickle cell disorders, connective tissue disorders, and coagulation defects compared to patients aged 35–44.9 and 45–54.9. They also experienced the longest hospital stays (2.08 days) and highest costs ($70,540). Significant odds ratios were found for sickle cell disorders (36.078), coagulation defects (1.566), inflammatory bowel disease (2.582), connective tissue disorders (11.727), hip dislocation (3.447), and blood transfusion (1.488) in younger patients compared to other THA patients. **Conclusions:** Comprehensive analysis of these unique needs is crucial for optimizing care, tailoring treatment, managing co-existing conditions, and personalizing recovery strategies to improve outcomes and quality of life for younger THA patients.

## 1. Introduction

Total hip arthroplasty (THA) has become a well-established surgical intervention for relieving pain and improving joint function in patients with severe hip conditions, particularly osteoarthritis [1,2,3,4]. Traditionally, THA has been performed primarily on older adults due to the gradual progression of degenerative joint diseases. However, recent years have witnessed a growing trend of THA procedures in younger patients [5,6]. This shift in demographics presents a unique set of challenges and considerations for orthopedic surgeons and healthcare providers managing this patient population.

Performing THA in younger patients has historically been approached with caution. Factors such as implant durability, increased polyethylene wear due to higher activity levels, and longer life expectancy have led to concerns about aseptic loosening and revision surgeries [5,7,8]. Despite these concerns, improvements in surgical procedures and implant materials have made THA feasible for young patients. Consequently, there has been an upward trend in the number of procedures performed on this age group over the past few decades [5,6,9].

The impact of age on THA outcomes presents a complex picture. While earlier studies reported promising results for younger patients [7], more recent research suggests higher rates of revision surgery and complications in this population [9,10,11]. This discrepancy might be due to increased functional demands placed on the implants by younger, more active individuals, coupled with their longer lifespans, leading to greater wear and tear [4,11]. Additionally, variations in study designs and patient populations make it challenging to definitively assess the long-term success of THA in younger patients.

The economic burden of THA on healthcare systems is significant. In the United States alone, hip arthroplasty accounted for a substantial number of hospitalizations and surgeries [12,13,14], highlighting its prevalence and associated costs. Understanding early postoperative complications is crucial, especially with the projected surge in THA demand fueled by an aging population and rising joint disease prevalence [12,13,14].

There is a significant lack of research on the clinical outcomes and healthcare usage patterns of younger patients receiving THA [1,2,7,12,13,14]. Most existing studies focus on older populations, leaving a critical gap in data on perioperative outcomes for younger individuals. This absence of information hinders effective clinical decision-making and resource allocation.

To address this knowledge gap, our study utilizes a nationwide big data analysis of the NIS database. We aim to explore the reasons for surgery (etiology), patient characteristics, and prevalence of comorbid conditions in younger THA patients (under 55 years old). Additionally, we will analyze hospital stays and postoperative complications across different age groups. This comprehensive analysis seeks to enhance the understanding of THA in younger patients, ultimately leading to better treatment strategies and healthcare policies.

### Research Questions

This study investigates the primary causes leading to THA in three younger age groups, examining the influence of demographics and clinical profiles on outcomes. It explores the prevalence and impact of comorbidities on postoperative complications and compares hospital stays, costs, and mortality rates across younger age subgroups. Additionally, it determines the odds ratios for various conditions and complications in younger patients compared to the overall THA population.

## 2. Methods

### 2.1. Data Source

The data were drawn from the Nationwide Inpatient Sample (NIS), a part of the Healthcare Cost and Utilization Project (HCUP) managed by the Agency for Healthcare Research and Quality (Rockville, MD, USA). The NIS provides a representative 20% stratified sample of all inpatient discharges from US hospitals, amounting to approximately seven million unweighted hospital stays annually. This study analyzed data from 1 January 2016 to 31 December 2019.

### 2.2. Study Population and Patient Variables

This study analyzed data for 231,630 adult patients (aged 18–54 years) who underwent total hip arthroplasty (THA). The patients were divided into three age groups: 18–34.9 years (*n* = 16,295), 35–44.9 years (*n* = 41,045), and 45–54.9 years (*n* = 174,290).

ICD-10 codes specific to THA procedures were used to identify patients. To ensure data accuracy, the study excluded patients admitted for emergency procedures, those with prior surgeries before admission, surgeries due to trauma, or surgeries related to oncology. A detailed list of ICD-10 codes for inclusion and exclusion is available in the Supplementary Materials.

Comorbidities were determined by reviewing each patient’s ICD-10 codes. Patients with hospital cost records of $0 were excluded. The study investigated the presence of the following comorbidities: hypertension, dyslipidemia, chronic anemia, osteoporosis, alcohol abuse, type 2 diabetes, renal disease, chronic heart failure (CHF), chronic lung disease, obesity, inflammatory bowel disease (IBD), coagulation defects, connective tissue disorders, sickle cell disease, and gout.

### 2.3. Outcome Measures

The study assessed several key outcomes for THA patients: in-hospital mortality, length of stay (LOS), complications, and total hospitalization costs. Established methods analyzed clinical outcomes. Specific ICD-10 codes identified complications like blood loss anemia, blood transfusion, and various other conditions. The postoperative period is defined as the time of the hospital stay.

### 2.4. Statistical Analysis

Statistical analysis using SPSS software version 26 involved calculating frequencies and proportions of factors like demographics, clinical characteristics, and hospital stay details. These were then weighted to reflect national estimates. Pearson’s chi-square test compared categorical variables; ANOVAs assessed differences in continuous variables. A *p*-value less than 0.05 indicated statistical significance.

### 2.5. Ethical Considerations

The study was conducted under exempt status granted by the institutional review board, and the requirement for informed consent was waived due to the de-identified nature of the NIS dataset.

## 3. Results

We focus on three age groups (16,295 patients 18–34.9 years old, 41,045 patients 35–44.9 years old, and 174,290 patients 45–54.9 years old), underlying causes, patient demographics, and outcomes like hospitalization length and complications. The mean age at admission for each group was as follows: 27.92 years (SD 5.358) for the under-35 group, 40.47 years (SD 2.755) for the 35–44.9 group, and 50.55 years (SD 2.712) for the 45–54.9 group, with a total mean age of 47.17 years (SD 7.180) for all groups combined.

### 3.1. Proportion of THA Procedures across Age Groups

**Figure 1:**
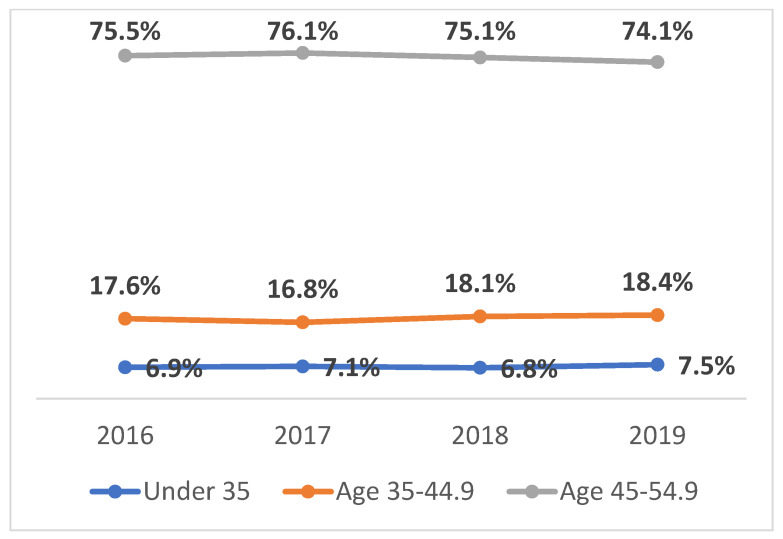
Percentage of THA procedures among different age groups (2016–2019).

From 2016 to 2019, the percentage of THA procedures performed on patients aged 18–34.9 increased from 6.8% to 7.5%, as shown in Figure 1. In numbers, this represents a slight increase each year: 4003 patients in 2016, 4081 in 2017, 4069 in 2018, and 4145 in 2019. The proportion of THAs in patients aged 35–44.9 showed slight fluctuations from 16.8% to 18.4%. Patients aged 45–54.9 consistently represented the largest proportion, ranging from 74.1% to 76.1% (*p* < 0.0001).

### 3.2. Etiologies of THA by Age Group

Primary osteoarthritis was the major cause of THA across all age groups. It accounted for 56.15% of cases in patients aged 18–34.9, increasing significantly to 77.30% in those aged 35–44.9, and 91.32% in the 45–54.9 age group (*p* < 0.0001). Osteonecrosis was more common in younger patients (35.86%) compared to older groups (20.16% and 7.77%). The chi-square test indicated that the distribution of etiologies for THA differed significantly among the three age groups (*p* < 0.0001). Specifically, etiologies such as congenital deformities of the hip, Legg–Calvé–Perthes disease, rheumatoid arthritis, juvenile rheumatoid arthritis, post-traumatic arthritis, acquired leg deformity, arthropathic psoriasis, and systemic lupus erythematosus were found to have a significantly different distribution across age groups. These conditions were more prevalent in the younger age group (18–34.9) compared to the older age groups (35–44.9 and 45–54.9). This statistical significance indicates that the etiologies for THA vary significantly among the three age groups, rather than implying that each etiology is significantly more prevalent in the youngest group (Table 1).

### 3.3. Demographics and Clinical Characteristics

The study revealed significant differences in demographics and clinical characteristics across age groups. Younger THA patients (18–34.9 years) were more likely to have Medicaid coverage (28.0%) compared to older patients. Conversely, private insurance prevalence increased with age, covering 56.8% of the youngest group, 65.2% of the 35–44.9 age group, and reaching 71.0% in the oldest patients (45–54.9 years). Racial makeup also differed by age. The youngest group had a higher proportion of Black (18.7%) and Hispanic (11.1%) patients compared to the older age groups. Pearson’s chi-square test was used to analyze the differences in demographics, clinical characteristics, and etiologies for THA among age groups. This test assessed the associations between categorical variables such as insurance type and racial composition across different age groups. The *p*-values indicating statistical significance are reported (Table 2).

### 3.4. Prevalence of Comorbidities in THA Patients by Age Group

Younger patients (18–34.9) had lower rates of hypertension (*p* < 0.0001) and dyslipidemia (*p* < 0.0001) compared to older patients. However, they exhibited higher rates of chronic anemia (*p* = 0.002) and inflammatory bowel disease (IBD) (*p* < 0.0001) compared to the oldest age group. Obesity (BMI over 35) and morbid obesity (BMI over 40) were more prevalent in the oldest age group (*p* < 0.0001 for both). The incidence of type 2 diabetes (*p* < 0.0001) and chronic lung disease (*p* < 0.0001) increased with age.

Younger patients also had a higher prevalence of sickle cell disorder (*p* < 0.0001) and coagulation defects (*p* < 0.0001) compared to older groups. The use of anticoagulants was highest in the oldest age group (*p* < 0.0001) (Table 3).

### 3.5. Hospitalization Outcomes by Age Group

According to Table 4, the LOS, total charges, and mortality rates varied by age group. Patients aged 18–34.9 years had the longest average stay (2.08 days) and the highest total charges ($70,540) compared to other age groups (*p* < 0.0001). Mortality rates remained low across all age groups, although statistically significant differences were observed (*p* = 0.009).

### 3.6. Postoperative Complications by Age Group

The rates of postoperative complications are presented in Table 5. Younger patients (18–34.9) had higher rates of blood loss anemia (16.69%), blood transfusion (3.80%), intraoperative fractures (0.83%), hip dislocation (0.49%), and pneumonia (0.15%) compared to older patients (*p* < 0.0001). Acute kidney injury and acute coronary artery disease increased with age.

### 3.7. Risk Estimates of Etiologies, Comorbidities, and Postoperative Complications

In this study, we compared three age groups; however, in this section, we focus on comparing patients under 35 with all other patients, encompassing a total of 1.6 million elective THA cases, to determine the odds ratios of various conditions and complications for younger patients compared to all ages of THA.

**Figure 2:**
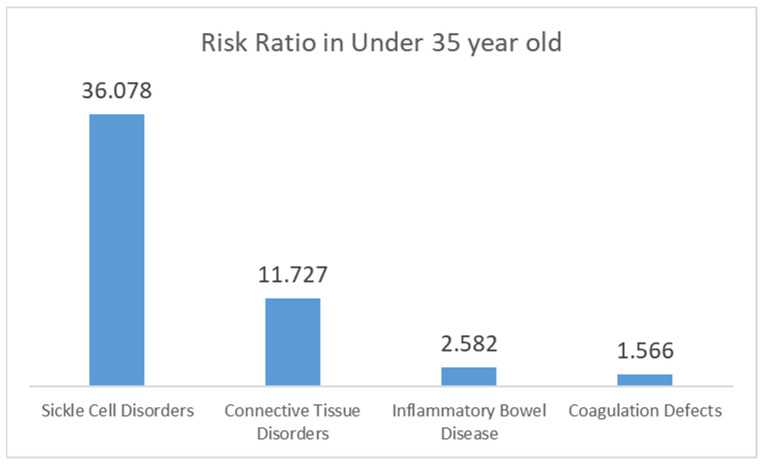
Odds ratios of etiologies and comorbidities in younger patients (under 35) undergoing THA compared to all other THA patients.

As shown in Figure 2, young patients with sickle cell disorders have an odds ratio of 36.078 (95% CI: 33.352–39.027), *p* < 0.0001. For coagulation defects, the odds ratio is 1.566 (95% CI: 1.360–1.802), *p* < 0.0001. IBD shows an odds ratio of 2.582 (95% CI: 2.270–2.938), *p* < 0.001, and connective tissue disorders have an odds ratio of 11.727 (95% CI: 9.470–14.523), *p* < 0.001.

**Figure 3:**
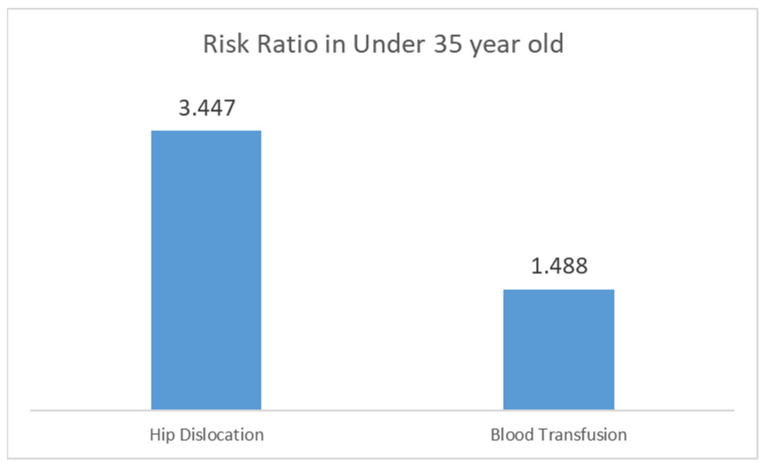
Odds ratios of postoperative complications in younger patients (under 35) undergoing THA compared to all other THA patients.

As shown in Figure 3, young patients have higher odds ratios for hip dislocation (3.447, 95% CI: 2.774–4.282, *p* < 0.001) and blood transfusion (1.488, 95% CI: 1.381–1.604, *p* < 0.001).

## 4. Discussion

While osteoarthritis remains the leading cause of THA across all ages, our study aligns with previous research by indicating a higher prevalence of secondary causes in younger patients [9,15,16]. These secondary causes, such as osteonecrosis, congenital deformities, post-traumatic arthritis, rheumatoid arthritis, and lupus, often arise from underlying health conditions or injuries rather than the age-related degeneration seen in older adults. This younger population undergoing THA frequently presents with complex medical histories and conditions that necessitate earlier intervention to preserve joint function and mobility [7,16].

Furthermore, younger THA patients exhibit a distinct comorbidity profile compared to their older counterparts. IBD, coagulation defects, connective tissue disorders, and sickle cell disease are more prevalent in this younger group. These conditions not only increase the risk of developing arthritis at a younger age but also complicate THA procedures and postoperative management [15,17,18,19,20,21]. The higher rates of blood loss anemia and blood transfusion needs observed in younger patients further highlight the challenges of managing bleeding during and after surgery in this population.

Our analysis revealed significant differences in postoperative complications across age groups. Younger patients have higher odds ratios for hip dislocation and blood transfusion, indicating a greater susceptibility to these complications compared to older patients. This aligns with existing literature documenting the increased risk of intraoperative and postoperative bleeding and the need for intensive management of these complications [22,23,24,25].

Our analysis revealed a clear trend: younger patients undergoing THA experienced longer hospital stays and incurred higher total charges compared to older patients [7,26,27]. This difference can be attributed to several factors. First, younger THA patients often have more complex medical histories, potentially requiring additional monitoring and interventions during their hospitalization. Second, the underlying cause of their arthritis might necessitate more extensive surgical procedures compared to older adults experiencing age-related degeneration. Finally, younger individuals tend to be more active after surgery. This higher activity level may require a more intensive rehabilitation program and a longer inpatient stay to ensure proper recovery.

These unique characteristics observed in younger THA patients highlight the importance of personalized surgical and postoperative care plans. Surgeons need to carefully consider the specific reasons for their arthritis (etiologies) and any pre-existing medical conditions (comorbidities) to optimize long-term outcomes.

For instance, the higher prevalence of coagulation defects in younger patients necessitates meticulous perioperative management of anticoagulation medications to minimize bleeding risks. Additionally, developing standardized protocols specifically tailored to younger patients with high post-surgical activity levels could improve rehabilitation outcomes and enhance patient satisfaction.

### Strengths and Limitations

This study delves into THA trends among younger patients by utilizing a large, nationally representative dataset. While this offers robust insights, the reliance on administrative data has limitations. It does not capture long-term outcomes or patient-reported measures [28,29,30,31], both crucial for a complete understanding of THA success in younger individuals. Future research should incorporate long-term follow-up data and patient-reported experiences to provide a more comprehensive picture of how THA impacts quality of life in this population. Despite these limitations, the study’s extensive data volume and long timeframe offer valuable information about demographics, comorbidities, early postoperative results, and the economic burden of THA in younger patients.

## 5. Conclusions

Understanding the unique challenges and outcomes associated with THA in younger patients is crucial for optimizing care and improving long-term results. By addressing specific etiologies, managing comorbidities effectively, and tailoring postoperative care, healthcare providers can enhance the success of THA in young patients under 55. This comprehensive approach will ultimately inform better treatment strategies and healthcare policies, ensuring improved outcomes and quality of life for younger THA patients.

## Figures and Tables

**Table 1:** Distribution of etiologies for THA by age group.

Etiologies	Under 35	Age 35–44.9	Age 45–54.9	Significance
Primary osteoarthritis (%)	56.15%	77.30%	91.32%	*p* < 0.0001
Osteonecrosis (%)	35.86%	20.16%	7.77%	
Congenital deformities of hip (%)	2.69%	1.14%	0.31%	
Legg–Calvé–Perthes disease	2.24%	0.34%	0.07%	
Rheumatoid arthritis (%)	1.15%	0.29%	0.21%	
Juvenile rheumatoid arthritis (%)	0.73%	0.11%	0.01%	
Post-traumatic arthritis (%)	0.60%	0.50%	0.20%	
Acquired leg deformity (%)	0.27%	0.11%	0.07%	
Arthropathic psoriasis (%)	0.12%	0.05%	0.03%	
Systemic lupus erythematosus (%)	0.18%	0.00%	0.01%	

**Table 2:** Patient demographics and clinical characteristics by age group.

Parameter	Under 35	Age 35–44.9	Age 45–54.9	Significance
Total surgeries (count)	16,295	41,045	174,290	-
Female (%)	45.4%	43.7%	46.1%	*p* < 0.0001
Payer—Medicare (%)	8.1%	9.2%	9.6%	*p* < 0.0001
Payer—Medicaid (%)	28.0%	19.1%	13.8%	
Payer—private (%)	56.8%	65.2%	71.0%	
Payer—other (including self-pay) (%)	7.2%	6.6%	5.6%	
Race—White (%)	62.5%	71.0%	78.2%	*p* < 0.0001
Race—Black (%)	18.7%	16.0%	13.0%	
Race—Hispanic (%)	11.1%	8.0%	5.4%	
Race—Asian or Pacific Islander (%)	2.9%	1.8%	1.0%	
Race—Native American (%)	0.6%	0.6%	0.4%	
Race—Other (%)	4.2%	2.7%	2.0%	

**Table 3:** Prevalence of comorbidities in THA patients by age group.

Parameter	Under 35	Age 35–44.9	Age 45–54.9	Significance
Hypertension diagnosis (%)	12.64%	28.15%	41.31%	*p* < 0.0001
Dyslipidemia diagnosis (%)	3.99%	13.03%	24.23%	*p* < 0.0001
Chronic anemia (%)	5.58%	4.98%	4.95%	*p* = 0.002
Osteoporosis (%)	1.26%	1.02%	1.32%	*p* < 0.0001
Alcohol abuse (%)	2.70%	3.16%	2.47%	*p* < 0.0001
Type 2 diabetes (%)	2.33%	6.42%	10.45%	*p* < 0.0001
Renal disease (%)	2.49%	1.89%	2.11%	*p* < 0.0001
CHF (%)	0.25%	0.32%	0.63%	*p* = 0.004
Chronic lung disease (%)	0.61%	2.07%	4.37%	*p* < 0.0001
Obesty (%)	18.35%	28.05%	30.42%	*p* < 0.0001
Morbid (severe) obesity (%)	9.79%	17.51%	18.62%	*p* < 0.0001
IBD	1.4%	1.1%	0.6%	*p* < 0.0001
Coagulation defects	1.2%	1.0%	0.8%	*p* < 0.0001
Connective tissues disorder	0.6%	0.3%	0.1%	*p* < 0.0001
Gout	0.7%	1.8%	2.5%	*p* < 0.0001
Use of anticoagulants	2.30%	2.05%	2.61%	*p* < 0.0001
Sickle cell disorder	5.3%	1.2%	0.3%	*p* < 0.0001

**Table 4:** Hospitalization outcomes by age group.

Parameter	Under 35	Age 35–44.9	Age 45–54.9	Significance
Died during hospitalization (%)	0.031	0.000	0.017	*p* = 0.009
Length of stay (mean in days)	2.08	1.89	1.82	*p* < 0.0001
Total charges (mean in $)	70,540	65,716	62,589	*p* < 0.0001

**Table 5:** Postoperative complications by age group.

Parameter	Under 35	Age 35–44.9	Age 45–54.9	Significance
Blood loss anemia (%)	16.69%	16.10%	15.25%	*p* < 0.0001
Blood transfusion (%)	3.80%	2.63%	2.04%	*p* < 0.0001
Intraoperative fracture (%)	0.83%	0.74%	0.66%	*p* < 0.0001
Hip dislocation (%)	0.49%	0.33%	0.30%	*p* < 0.0001
Pneumonia (%)	0.15%	0.05%	0.09%	*p* < 0.0001
Venous thromboembolism (%)	0.12%	0.16%	0.07%	*p* < 0.0001
Pulmonary embolism (%)	0.09%	0.11%	0.06%	*p* = 0.005
Acute kidney injury (%)	0.61%	0.69%	0.81%	*p* = 0.002
Acute coronary artery disease (%)	0.00%	0.00%	0.02%	*p* = 0.003
Pulmonary edema (%)	0.00%	0.00%	0.02%	*p* = 0.001
Heart failure (%)	0.00%	0.01%	0.01%	*p* = 0.301

## Data Availability

The original contributions presented in the study are included in the article/Supplementary Materials, further inquiries can be directed to the corresponding author.

## Supplementary Materials

The following supporting information can be downloaded at https://www.mdpi.com/article/10.3390/jcm13154535/s1: Table S1: ICD10 codes.

## Abbreviations

HCUP	Healthcare Cost and Utilization Project
IBD	Inflammatory bowel disease
ICD-10	International Classification of Diseases, 10th Revision
LOS	Length of stay
NIS	Nationwide Inpatient Sample
SPSS	Statistical Package for the Social Sciences
THA	Total hip arthroplasty
