# Supplementary material for: Etiology, Outcomes, and Complications of Total Hip Arthroplasty in Younger Patients: A Nationwide Big Data Analysis
Source: J Clin Med. 2024 Aug 2;13(15):4535. doi: 10.3390/jcm13154535 (PMC11313375; doi:10.3390/jcm13154535)
Supplement: Supplementary file 1 [file jcm-13-04535-s001.zip › jcm-3103834-supplementary.pdf]

Table S1: ICD10 codes.

ICD 10 CODES / PROCEDURE CODE	
0SRC069, 0SRC06A, 0SRC06Z, 0SRC07Z, 0SRC0J9, 0SRC0JA, 0SRC0JZ, 0SRC0KZ, 0SRC0L9, 0SRC0LA, 0SRC0LZ, 0SRD069, 0SRD06A, 0SRD06Z, 0SRD07Z, 0SRD0J9, 0SRD0JA, 0SRD0JZ, 0SRD0KZ, 0SRD0L9, 0SRD0LA, 0SRD0LZ	Total knee arthroplasty procedure
8E0Y0CZ,8E0YXCZ	Robotic Assisted Procedure of Lower Extremity
I5021, I5031, I5033, I5041, I5043	Heart Failure
N170, N171, N172, N178, N179	Acute Kidney Injury
I2101, I2102, I2109, I211, I2119, I2111, I212, I2129, I213, I214, I219	Acute Coronary Artery Disease
I60, I61, I62, I63, I650, I688, O873, O2250, O2251, O2252	Stroke
J810, J811, I501	Pulmonary Edema
I10(start with)	Hypertension
D62 (start with)	Blood Loss Anemia
J189, J159, J22	Pneumonia
I2602, I2609, I2692, I2699	Pulmonary Embolism
I82401, I82402, I82403, I82409, I82411, I82412, I82413, I82419, I82421, I82422, I82423, I82429	DVT
E78(start with)	Dyslipidemia
G473	Obstructive Sleep Apnea
D64(start with)	Chronic Anemia
F10	Alcohol Abuse History
M81, M82	Osteoporosis
F (start with)	Mental Disorders
G20 (start with)	Parkinson Disease
E11 (start with)	Type 2 Diabetes Mellitus
N18 (start with)	Chronic Kidney Disease
I500, I501, I509	Congestive Heart Failure
J44 (start with)	Chronic Lung Disease
K50 (start with) and K51 (start with)	IBD
Q874 (start with) and Q796 (start with)	Connective tissues disorder
M10 (start with)	Gout
D68 (start with)	Coagulation defects
